# Editorials

**Published:** 1914-02

**Authors:** 


### EDITORIAL

The Business Office of the *Journal-Record* is 23 West Alabama Street.

The Editorial Office is 1014-15 Atlanta National Bank Building.

Address all Business Communications to *Journal-Record of Medicine*, 23 West Alabama Street.

Make remittances payable to *The Journal-Record of Medicine.*

On matters pertaining to the Editorial and Original Communications, address Edgar G. Ballenger, M. D., Atlanta, Ga.

Reprints of Original Articles will be furnished at cost price. Requests for the same should always be made in THE MANUSCRIPT.

We will present, postpaid, on request to each contributor of an original article, twenty (20) marked copies of *The Journal-Record of Medicine* containing such article.

### "FOLLOW UP."

Dr. Richard C. Cabot, of the Massachusetts General Hospital, has applied this idea to medicine as business men use it in control of their trade and the extension of their interests. He has shown the great waste that accrues to the purely clinical work of diagnosis and immediate treatment when the patient is not observed in the home later on. His speeches and writings are chiefly applicable in their teaching to the patients that come to a hospital or clinic, but the principle may well be applied to the private practice of the physician who enters the homes of his patients.

When a child is suffering with sickness incident to improper feeding, the diet prescribed is carefully attended to by the parents, but all the time it is somewhat of a burden and the mother longs for the day when the regular diet of the family may be sufficient for every member of it. And, sooner or later, that kind of food is substituted for the viands upon which recovery was based. In good time another attack arrives, and the old round is gone through with again. A bit of follow-up by the family doctor who can enter the homes of his families almost when he likes, would have given him a chance to mark the faulty food and give the warning that would protect not only the ailing child, but the adult members as well, from the strain the food imposed.

The same thing might well be said of clothing, methods of sleeping, habits of bathing and almost numberless other things in hygiene and sanitation, as well as the special causes of particular diseases the family may have had to withstand.

At the time of the illness, it might not be clear what led up to the trouble, but later when the usual habits and methods are resumed, the cause of the trouble is often strikingly apparent.

Not only will rectification of untoward conditions prevent disease, but it inevitably increases general comfort, once the change is instituted, and the healthy, together with the weak, will have increase of happiness.

The man who does his daily work in the homes has a great opportunity for good in his follow-up observations and advice, and he may be sure that his reputation as well as his purse will increase with the growth of confidence in his judgment among his people as they realize the beneficent results of his guidance.

